# Herbivore species asynchrony underpins multitrophic stability across plants, herbivores and predators

**DOI:** 10.1111/1365-2656.70318

**Published:** 2026-07-18

**Authors:** Takehiro Sasaki, Taiki Tachibana, Xiaoming Lu, Kei Uchida, Xuezhen Zhao, Issei Nishimura, Daichi Makishima, Kousuke Tachibana, Xi Sun, Yuki Iwachido, Wenhuai Li, Yongfei Bai

**Affiliations:** ^1^ Department of Natural Environment Graduate School of Environment and Information Sciences, Yokohama National University Yokohama Kanagawa Japan; ^2^ Institute for Multidisciplinary Sciences, Yokohama National University Yokohama Japan; ^3^ State Key Laboratory of Vegetation and Environmental Change Institute of Botany, Chinese Academy of Sciences Beijing China; ^4^ Faculty of Environmental Studies Tokyo City University Yokohama Japan; ^5^ Ministry of Education Key Laboratory of Ecology and Resource Use of the Mongolian Plateau & Inner Mongolia Key Laboratory of Grassland Ecology & Observation and Research Station for the Typical Steppe Ecosystem of the Ministry of Education School of Ecology and Environment, Inner Mongolia University Hohhot China

**Keywords:** biodiversity, compensatory dynamics, ecosystem functioning, insurance hypothesis, mass ratio hypothesis, multitrophic stability, removal experiment, species synchrony

## Abstract

1. Ecological stability is fundamental to sustaining ecosystem functioning amid accelerating global environmental change. Both theoretical and empirical evidence suggest that biological diversity and composition influence species asynchrony and species stability within individual trophic levels, thereby contributing to greater temporal stability at the community level. Nonetheless, it remains unclear whether and how these stabilizing factors are integrated across trophic levels to determine multitrophic stability.
2. In this study, using 3 years of community data collected approximately 10 years after the manipulation of plant diversity in a grassland biodiversity experiment, we used structural equation models to assess how species richness, functional composition, species asynchrony and species stability within plant, herbivore and predator communities were associated with multitrophic stability. We quantified multitrophic stability in two complementary ways: an averaging approach, based on the overall mean stability across trophic levels, and a multiple‐threshold approach, based on how many trophic levels maintained high stability concurrently. This framework allowed us to examine whether key predictors of multitrophic stability were shared between the two metrics or differed depending on the metric used.
3. Asynchrony among species within each trophic group typically contributed to enhanced community stability. At the multitrophic level, analyses using the averaging approach revealed that average multitrophic stability was positively associated with herbivore species asynchrony and richness, predator functional composition and predator species asynchrony, with herbivore species asynchrony showing a particularly strong positive relationship. Similarly, employing the multiple threshold approach, which evaluated how many trophic levels exceeded 50%, 60%, 70% or 80% of the maximum stability, further identified herbivore species asynchrony as a significant positive predictor at 70% and 80% stability thresholds. Herbivore species richness, by contrast, showed threshold‐dependent effects, with positive indirect effects at the 70% and 80% thresholds but negative direct effects at the 50% and 70% thresholds, potentially attributable to diminished plant community stability.
4. Our findings suggest that multitrophic stability across plants, herbivores and predators is most closely associated with the compensatory dynamics of the intermediate consumer trophic level—specifically, herbivores—highlighting this trophic level as a key contributor to buffering temporal fluctuations in multitrophic communities.

## Graphical abstract

Across a grassland food web, multitrophic stability was most closely associated with herbivore species asynchrony. By identifying herbivores as a key source of temporal buffering between plants and predators, this study shows that intermediate consumers can play a central role in stabilizing the complex food web.

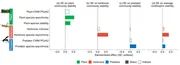

## 1 INTRODUCTION

Substantial efforts have been made to explore the drivers of community stability (Eisenhauer et al., 2024; Isbell et al., 2015; Tilman et al., 2006; Valencia et al., 2020), often conceptualized as the temporal invariability (i.e. inverse of the coefficient of variation) of aggregate properties, such as total biomass or abundance (Liang et al., 2022; Meng et al., 2023; Sasaki et al., 2024). Two components are particularly crucial among these dynamics: species asynchrony—the extent to which different species fluctuate independently over time—and species stability—which refers to the temporal stability of constituent species weighted by their abundance in the community (Loreau et al., 2021; Loreau & de Mazancourt, 2008; Sasaki & Lauenroth, 2011; Smith et al., 2020; Wilcox et al., 2017; Yachi & Loreau, 1999). Theoretical and empirical evidence suggest that biological diversity and composition can influence species responses to environmental changes, dominance patterns and resource use (Firkowski et al., 2022; Loreau et al., 2021; Sasaki et al., 2019; Scherber et al., 2010). These influences shape species asynchrony and species stability, ultimately affecting the overall community stability (Loreau et al., 2021; Loreau & de Mazancourt, 2008; Sasaki et al., 2019; Wilcox et al., 2017; Yachi & Loreau, 1999).

Historically, most research on the relationship between biodiversity and stability has focused on a single trophic level, often reporting that greater species diversity and asynchrony tend to stabilize plant communities. However, more recent empirical studies have expanded our understanding by highlighting the importance of multitrophic interactions in maintaining ecosystem stability (Liang et al., 2025; Srednick & Swearer, 2024). For instance, the loss of consumer groups such as arthropods or fungi can destabilize plant communities by reducing asynchrony among plant species (Liang et al., 2025). Moreover, Srednick and Swearer (2024) advanced diversity–synchrony–stability research beyond single trophic levels by examining these relationships in an algal–herbivore system. However, their study focused on a two‐trophic‐level system and primarily relied on an aggregate metric of multitrophic stability, leaving it unclear how factors associated with stability within individual trophic levels scale up to shape multitrophic stability in more complex food webs.

A key unresolved question is whether the stabilizing factors within individual trophic levels also maintain community stability across interconnected trophic levels. This scaling is not straightforward because adjacent trophic levels are linked through resource quantity and quality, prey availability and consumer pressure (Scherber et al., 2010; Schmitz, 2008; Thébault & Loreau, 2005). Plant diversity and functional composition can influence herbivore communities by altering the amount, quality and temporal heterogeneity of plant resources available to consumers (Loranger et al., 2012; Lu et al., 2022; Scherber et al., 2010), whereas herbivore diversity and functional composition can influence predator communities by altering prey availability and prey trait composition (deCastro‐Arrazola et al., 2023; Firkowski et al., 2022). Effects between non‐adjacent trophic levels are expected to be mediated primarily through the intermediate trophic level rather than through direct plant–predator linkages (Schmitz, 2008; Thébault & Loreau, 2005). Yet, most studies have not clearly examined how factors associated with community stability within trophic levels scale up to broader multitrophic stability. With global changes increasingly impacting multitrophic biodiversity (Barnes et al., 2017; van Moorsel et al., 2023), it has become imperative to understand the core principles underlying multitrophic stability.

In this study, we investigated species dynamics and community stability at three distinct trophic levels—plants, herbivores and predators—to determine their roles in maintaining overall multitrophic stability. To this end, we explored factors associated with multitrophic stability through a biodiversity experiment in a natural grassland in Inner Mongolia (Sasaki et al., 2019; Wu et al., 2015). Using time‐series data from plants, herbivores and predators, we assessed how species richness, functional diversity, functional composition, species asynchrony and species stability at each trophic level contribute to single‐trophic‐level community stability and to multitrophic stability (Figure S1).

For the stability of each trophic‐level community (Figure S1a–c), we expected species richness, functional diversity and functional composition within each trophic group to influence species asynchrony and species stability by altering dominance structure, trait composition and resource partitioning among constituent species (Craven et al., 2018; Sasaki et al., 2019; Schnabel et al., 2021). Species asynchrony was expected to enhance single‐trophic‐level community stability through compensatory fluctuations among species, whereas species stability was expected to contribute to it through greater temporal stability of constituent species (Eisenhauer et al., 2024; Sasaki et al., 2019; Tilman et al., 2006; Valencia et al., 2020; Wilcox et al., 2017). We also hypothesized species richness, functional diversity and functional composition to contribute directly to single‐trophic‐level community stability, because trait composition and dominance structure may affect aggregate community variability beyond their effects on species asynchrony and species stability alone (Craven et al., 2018; Sasaki et al., 2019). In addition, as adjacent trophic levels can most directly alter resource supply or consumer pressure, we expected species asynchrony and species stability in neighbouring trophic groups to be associated with focal community stability (Schmitz, 2008; van Veen et al., 2006).

For multitrophic stability (Figure S1d), we tested whether species asynchrony and species stability within plants, herbivores and predators were associated with multitrophic stability, rather than assuming that stability components important within individual trophic levels would necessarily scale up across trophic levels. We also evaluated direct relationships among species richness, functional diversity and functional composition at each trophic level and multitrophic stability, recognizing that these variables may reflect potential pathways through which trophic structure impacts food web stability.

To evaluate multitrophic stability, we considered two complementary approaches: the averaging approach (Maestre et al., 2012) and the multiple threshold approach (Byrnes et al., 2014). The averaging approach summarizes mean stability across trophic levels, though it can mask differences in how stability is distributed among trophic groups—similar averages may arise either when all trophic levels show moderate stability or when some trophic levels are highly stable whereas others are unstable (Byrnes et al., 2014). In contrast, the multiple threshold approach evaluates the extent to which stability persists across all trophic levels. These approaches can, therefore, capture two complementary aspects of multitrophic stability: the overall mean level of stability across trophic levels and the simultaneous maintenance of high stability across trophic levels. Using this framework, we asked whether diversity, functional composition and stability components across plants, herbivores and predators showed similar associations with these two complementary measures of multitrophic stability or whether the key predictors differed between them.

## 2 MATERIALS AND METHODS

### 2.1 Experimental site

This study was conducted in the Xilin River Basin, Inner Mongolia, China (43°33′ N, 116°41′ E; elevation: 1275 m), managed by the Inner Mongolia Grassland Ecosystem Research Station (Chinese Academy of Sciences). From 2000 to 2016, on‐site weather station data showed that the region had a semi‐arid climate, with an average annual temperature of 0.9°C and 301 mm of precipitation. Precipitation from January to July (mean: 179 mm) has been identified as the key climatic driver influencing the structure and function of this grassland ecosystem (Bai et al., 2004). The soil is dark chestnut (Calcic Chernozem), loamy‐sand textured. Vegetation primarily comprises *Leymus chinensis* (perennial rhizomatous grass) and *Stipa grandis* (perennial bunchgrass), along with other grasses (e.g. *Cleistogenes squarrosa* and *Agropyron cristatum*) and forbs (e.g. *Allium tenuissimum* and *Artemisia frigida*) at lower abundances (Bai et al., 2004).

### 2.2 Experimental design

The Inner Mongolia Grassland Removal Experiment (IMGRE), initiated in 2005, is a field manipulation study investigating how plant diversity affects ecosystem functioning (Wu et al., 2015). Although the full experiment included eight blocks (55 × 85 m each), the current study used four blocks to streamline sampling. Each block comprised 32 plots (6 × 6 m), resulting in a total of 128 plots analysed (*n* = 128).

IMGRE targets four main plant functional groups (PFGs): perennial rhizome grasses (PR), perennial bunch grasses (PB), perennial forbs (PF) and annuals and biennials (AB). Shrubs and sub‐shrubs were excluded due to their scarcity. All 16 possible combinations of PFG removals were implemented using a randomized block design, which enabled a full factorial treatment structure.

Two protocols were adopted to manipulate plant diversity and control disturbance effects: complete and partial removal. For complete removal, all individuals of the targeted PFGs were clipped annually from 2006 to 2009 at soil depths of 0–3 cm, creating a discrete gradient, with plots retaining 0–4 PFGs. In the partial removal protocol, ~50% of vegetation was removed using a quadrat‐based method (Sasaki et al., 2019), generating a continuous diversity gradient while ensuring similar disturbance intensity across treatments.

To minimize the impact of initial disturbances, such as changes in resources or soil structure, natural plant communities were allowed to reassemble after the last removal in 2009. This extended recovery period helped reduce short‐term disturbance legacies (Díaz et al., 2003; Grman et al., 2010) and encouraged persistent variation in plant diversity and functional trait composition (i.e. community‐weighted means [CWMs]) under relatively uniform pretreatment conditions (Sasaki et al., 2019; Wu et al., 2015).

The experimental design was originally intended to generate variation in plant diversity and functional composition under relatively uniform pretreatment conditions, thereby mimicking realistic biodiversity‐loss scenarios under global change, including intensive grazing or extreme drought (Lisner et al., 2023; Sasaki et al., 2017; Smith & Knapp, 2003). However, because plant and arthropod communities were surveyed only after the final removals in 2009 and following approximately 10 years of natural reassembly, the present communities should not be interpreted as reflecting strong persistent treatment effects alone. Rather, they represent communities that reassembled under different treatment histories after a long recovery period. Nonetheless, this system provides a useful platform for examining how variation in species richness, functional diversity and functional composition among plants, herbivores and predators is associated with multitrophic stability.

To determine whether variation associated with these different treatment histories in plant community characteristics was still expressed during sampling, we compared mean plant richness, functional diversity and functional composition (CWM PCoA1 and CWM PCoA2; see Section 2.6), averaged across 2017–2019, between removal treatments and control plots using linear mixed‐effects models with treatment history as a fixed effect and block as a random effect, analysed separately for the complete and partial removal protocols (Table S1; Figure S2). These analyses showed among‐plot variation in plant community properties during 2017–2019 following long‐term reassembly (Figure S2); because differences from control plots were not consistently significant, this variation should not be interpreted as evidence for persistent effects of the original treatments.

### 2.3 Plant and arthropod community composition

In mid‐August from 2017 to 2019—coinciding with peak plant biomass (Bai et al., 2004)—above‐ground biomass was assessed by harvesting all vegetation at the soil surface within a 1 × 1 m quadrat in each plot. To mitigate the impact of repeated harvesting, the quadrat location was randomly repositioned within each plot at each sampling event. All plant material was identified and sorted by species, oven‐dried at 65°C for 48 h and weighed to determine biomass.

Arthropod sampling was conducted on clear, windless days from mid‐July to mid‐August between 2017 and 2019 (once per plot per method annually), employing three complementary approaches to comprehensively capture herbivorous and predatory taxa. Sweep net sampling (42 cm diameter, 1.5 m length) was conducted at each plot corner by 4 observers, each performing 300 sweeps in separate, non‐overlapping areas, resulting in 1200 sweeps per plot.

Flight intercept traps (FITs; intercept panels 45 × 30 cm) were positioned at the centre of each plot for 24 h. Each trap comprised two transparent acrylic panels (30 × 45 cm) arranged in a cross configuration, mounted between circular trays (30 cm diameter, 5 cm height). The lower tray contained water mixed with surfactant to retain insects that collided with the panels and fell into the trap (Southwood & Henderson, 2009). As the maximum vegetation height at the site was 40–50 cm, panel heights overlapped with the main flight layer of grassland arthropods.

Pitfall traps (7 cm diameter, 12 cm depth) were installed at three corners, 1 m from the plot edge and left for 24 h to collect ground‐dwelling arthropods. While arthropods may still enter from outside the plots, traps were placed identically across all plots to reduce bias.

Sampling times were standardized across plots: sweep netting was performed from 09:00 to 12:00 and 14:00 to 17:00 to avoid midday heat; FITs were deployed from 08:00 to 9:00 and retrieved from 17:00 to 18:00; and pitfall traps were set during the day and retrieved the following day. To avoid interference among methods in arthropod sampling, each method was conducted on separate days, with a minimum interval of 3 days.

Collected specimens were fixed in ethyl acetate and sorted by the sampling methods. As species‐level identification was often impractical, individuals were classified into morphospecies based on morphological traits and feeding habits, aided by available literature (Lu et al., 2021, 2022) and input from taxonomic experts. Based on these resources, arthropods were classified as either herbivores or predators. For data analysis, the results from the sampling techniques were combined into a single species‐by‐plot matrix for each year.

### 2.4 Plant traits

A total of 21 locally common plant species were selected for trait measurement. Leaf and root traits (Table S2) were assessed using standardized protocols in five ungrazed areas, each located at least 50 m apart and near the experimental plots in August 2013 (Pérez‐Harguindeguy et al., 2013). Species with a relative abundance less than 1% at the plot level were excluded. The selected 21 species collectively represented approximately 99.1 ± 2.9% of the total community biomass (mean ± SD across plots and years during 2017–2019), effectively capturing the dominant species within these communities.

Recognizing fundamental differences between plants and arthropods in trait dimensions governing ecological strategies and trophic interactions, trophic group‐specific trait sets were selected to capture the main functional axes within each group. For plants, these axes include resource acquisition and conservation, along with size‐related variation linked to competitive ability, light capture and niche differentiation (Craven et al., 2018; Lavorel et al., 2011; Pérez‐Harguindeguy et al., 2013; Sasaki et al., 2019; Sperandii et al., 2025). These trait dimensions can influence plant growth, reproduction, survival and responses to environmental variability and are thus expected to contribute to temporal community dynamics and stability.

For each species, 16 plant traits were assessed (Table S2), representing key dimensions of plant functional strategy (Sasaki et al., 2019). In each area, at least nine fully expanded leaves were collected from at least three mature individuals per species. Plant height (cm) was defined as the distance from the base to the tip of the highest leaf and was measured on the same individuals. Leaf area (cm^2^) was determined from scanned leaves using ImageJ (ver. 1.48). Fresh and dry masses (g) were measured after water saturation and oven‐drying; the resulting values were used to calculate specific leaf area (SLA; cm^2^ g^−1^) and leaf dry matter content (LDMC; g g^−1^). Leaf tensile strength (N cm^−1^) was measured using a force gauge (DS2‐5N, IMADA Co., Ltd., Aichi, Japan). Composite samples per area were analysed for leaf carbon, nitrogen and phosphorus content (g g^−1^).

For the same individuals, fine root morphology was measured, including root diameter (cm), specific root length (cm g^−1^) and fine root carbon, nitrogen and phosphorus content (g g^−1^). Fine roots (<2 mm diameter) were carefully washed from excavated root systems, and root diameter and specific root length were measured following standard protocols. Seed mass (mg) was estimated from seeds collected from 30 individuals per species across areas. Trait values used in the analyses were calculated as species‐level means averaged across the five sampling areas. The resulting matrix of 16 plant traits by 21 species was used in subsequent analyses.

### 2.5 Arthropod traits

Morphological traits were assessed for arthropod species (Table S2) using a minimum sample size of five individuals, collected during the arthropod community composition survey (see Section 2.3) in August 2017 and 2018, following standardized protocols (Moretti et al., 2017). The evaluated traits comprised dry weight (g), body size (cm), wing size (cm) and body hairiness (unitless). These characteristics were selected to represent key functional axes likely to influence trophic interactions and temporal dynamics within consumer communities. Specifically, body size and dry weight serve as established proxies for trophic role, resource availability and per‐capita consumption potential, whereas wing size provides an index of mobility and dispersal capacity. Body hairiness pertains to defence mechanisms and interaction‐related functions (deCastro‐Arrazola et al., 2023; Moretti et al., 2017). Given their impact on resource exploitation, predation avoidance and trophic‐level interactions, these traits are considered particularly relevant to the dynamics and stability of multitrophic communities. Dry weight, body size and wing size were recorded after the specimens were treated with ethyl acetate and air‐dried on absorbent cotton for at least 2 weeks. Body hairiness was evaluated using a five‐point scale (1–5), where 1 denotes the absence of visible hair and 5 indicates dense hair coverage, based on visual assessment of the ventral, dorsal and leg surfaces. Scores were averaged across body regions.

### 2.6 Functional diversity and composition at each trophic level

To quantify functional diversity within each plot for plants, herbivores and predators, we calculated functional dispersion (FDis; Laliberte & Legendre, 2010) using a Gower dissimilarity matrix based on the set of traits (Table S2). FDis calculations were weighted according to relative abundance—above‐ground biomass for plants and individual counts for herbivores and predator arthropods. FDis was calculated separately for each plot and year, and the resulting values were averaged across 2017–2019 for the following analyses.

Additionally, we independently calculated CWM trait values (Garnier et al., 2004) for plants, herbivores and predators to characterize the dominant traits in each plot per year. These CWM values were averaged across 2017–2019 for the following analyses. To reduce the dimensionality of the trait space across these groups, principal coordinate analysis (PCoA) was conducted and species coordinates along the first two axes were used as synthetic trait values following established methodologies (Bishop et al., 2015; Villéger et al., 2013).

For plants, we interpret the first two PCoA axes as composite trait gradients rather than as pure representations of single ecological strategies, because whole‐plant trait spaces are typically multidimensional (Díaz et al., 2016; Weigelt et al., 2021). The first PCoA axis was positively associated with specific leaf area, leaf nitrogen content and root carbon content and negatively associated with leaf carbon content (Figure S3a). We therefore interpret PCoA1 as a composite gradient contrasting relatively acquisitive leaf syndromes with more carbon‐rich, structurally invested syndromes. This interpretation is consistent with reports that leaf nitrogen and leaf carbon concentrations can vary in opposite directions among species (Zhou et al., 2022) and that leaf carbon concentration may be related to leaf structural investment and construction traits (Xing et al., 2021). The second axis was positively associated with specific fine root length and negatively associated with root diameter, root nitrogen and phosphorus concentrations, leaf area and plant height. Therefore, we do not interpret PCoA2 as a single below‐ground resource‐use gradient. Instead, we describe it as a composite trait axis capturing covariation, in this dataset, among fine‐root morphology, root nutrient concentrations and plant size‐related traits. This cautious interpretation is consistent with the multidimensional view of fine‐root trait coordination, in which morphological and nutrient‐related root traits do not necessarily vary along a single acquisitive–conservative axis (Bergmann et al., 2020; Kramer‐Walter et al., 2016; Weemstra et al., 2016).

In herbivores and predators, the first PCoA axis was most strongly associated with body size, while the second axis corresponded to hairiness (Figure S3b,c). These axes relate to resource use and prey availability in herbivores and to prey defence and predators' efficiency, respectively.

### 2.7 Community stability at single and multitrophic levels

Temporal stability within plant, herbivore and predator communities was assessed by calculating the ratio of the temporal mean to the SD of plant above‐ground biomass, herbivore abundance and predator abundance, respectively. Annual data from 2017 to 2019 were utilized for this purpose. Abundance served as the metric for arthropods, as estimating biomass for diverse, small‐bodied species is impractical, and abundance is widely recognized as an effective proxy for community dynamics in arthropod research (Scherber et al., 2010). Given that each plot's time series comprised only three annual observations, we did not detrend plant biomass, herbivore abundance or predator abundance before calculating stability. Linear trend estimation and residual variance analysis are statistically unreliable and highly susceptible to noise with such limited data. Accordingly, temporal stability was determined using the raw SD for each community variable. To improve normality, temporal stability at each trophic level was log‐transformed using the natural logarithm. This relative measure of invariability, scaled by the mean, is widely used in studies investigating biodiversity–stability relationships (Hautier et al., 2020; Liang et al., 2022; Meng et al., 2023; Sasaki et al., 2024; Tilman et al., 2006; Valencia et al., 2020), facilitating comparability with the existing literature.

### 2.8 Species asynchrony and species stability at each trophic level

Species richness, species asynchrony and species stability were determined independently for plants, herbivores and predators. For each trophic level, species richness was defined as the mean number of plant, herbivore and predator species per plot across the observation years. Species asynchrony (1 − *φ*) for each plot was calculated using Equation (1) (Loreau & de Mazancourt, 2008):

(1) $$1-\varphi =1-\sigma^{2}/{\left(\sum_{i=1}^{S}\sigma_{i}\right)}^{2}$$

where *σ* ^2^ is the temporal variance of total community biomass (plants) or abundance (herbivores or predators) in each plot from 2017 to 2019, while *σᵢ* is the temporal standard deviation (SD) of biomass (plants) or abundance (herbivores or predators) for species *i* across the same years, with *S* being the number of species. Species asynchrony ranges from 1 (perfectly asynchronized species fluctuations) to 0 (perfectly synchronized species fluctuations) (Loreau & de Mazancourt, 2008). Species stability per plot was calculated as the sum of each species' temporal stability, weighted by their relative abundance over time (Meng et al., 2023; Sasaki et al., 2024; Wilcox et al., 2017).

### 2.9 Data analysis

Community stability was initially evaluated independently for plants, herbivores and predators. Subsequently, multitrophic stability was assessed using two complementary approaches. The averaging approach (Maestre et al., 2012) involved standardizing the community stability of each trophic level to a 0–1 scale, then calculating the average multitrophic stability for each plot. This approach was used to summarize the overall mean stability across trophic levels. In contrast, the multiple threshold approach (Byrnes et al., 2014) quantified, for each plot, the number of single‐trophic‐level community stability metrics that exceeded a defined threshold. These approaches captured complementary aspects of multitrophic stability; however, neither explicitly evaluates nor quantifies potential trade‐offs or synergies among trophic levels.

Thresholds were defined as percentages of the maximum observed stability at each trophic level, calculated as the mean stability value above the 95th percentile to mitigate the impact of outliers. Similar outlier‐robust near‐maximum definitions are widely used in threshold‐based multifunctionality studies (Byrnes et al., 2014; Cruz‐Alonso et al., 2019; Sasaki et al., 2019; van der Plas et al., 2019). For multiple threshold analyses, representative moderate‐to‐high thresholds—50%, 60%, 70% and 80% of the maximum observed stability—were selected to identify high‐stability conditions while maintaining a clear and consistent presentation of SEM results (Chen et al., 2023; Sasaki et al., 2022). Thresholds above 85% were excluded from the analysis because model convergence could not be achieved due to limited variation in the response variable.

Structural equation modelling (SEM) was applied to assess how species richness, functional diversity, functional composition (CWMs of PCoA axes), species asynchrony and species stability across plants, herbivores and predators were related to (i) the community stability of each trophic group (i.e. single‐trophic‐level community stability; Figure S1a–c) and (ii) average multitrophic stability (Figure S1d). To prevent multicollinearity, we excluded predictors with high correlations (Pearson's |*r*| >0.70; Table S3), resulting in the exclusion of FDis and CWM PCoA2 for herbivores and CWM PCoA1 for predators. We included correlated errors among all pairs of diversity and composition variables within each trophic level, as well as between all pairs of stability‐component variables within the same and adjacent trophic levels (Figure S1). To account for the nested structure of the treatments and removal protocols within blocks, we used the svydesign function from the survey package and the lavaan.survey function from the lavaan.survey package in R. We simplified the SEM by sequentially removing non‐significant paths (*p* > 0.05) until the most parsimonious model was obtained. Model fit was evaluated using the Bollen–Stine bootstrap test (Bollen & Stine, 1992), using 1000 bootstrap samples to correct for non‐normal data. A Bollen–Stine *p*‐value >0.10 indicated a good fit. Standardized total effects were calculated from the fitted SEM to quantify the overall influence—both direct and indirect—of predictors on community stability.

For threshold‐based multitrophic stability, the same SEM framework was applied at 50%, 60%, 70% and 80% thresholds, beginning with the same set of predictors for each threshold (Figure S1d). These models facilitated the evaluation of how species richness, functional diversity, functional composition, species asynchrony and species stability across plants, herbivores and predators relate to the number of trophic‐level stability metrics that exceed each threshold. As each threshold defines a different version of the response variable, the structure of the best‐fitting SEM may differ among thresholds. We therefore summarized standardized effects separately for these representative thresholds, rather than presenting them as continuous threshold profiles, as suggested by previous studies (Chen et al., 2023; Sasaki et al., 2022).

All data analyses were conducted using R version 4.4.1 (R Development Core Team, 2024). The ‘FD’ package was employed to calculate functional diversity metrics and CWM trait values (Laliberté et al., 2014), while ‘lme4’ was used to fit linear mixed‐effects models (Bates et al., 2015). For community stability metrics, the ‘codyn’ package was utilized (Hallett et al., 2016). SEM analyses were performed with the ‘lavaan’ (Rosseel, 2012), ‘lavaan.survey’ (Oberski, 2014) and ‘survey’ (Lumley & Lumley, 2020) packages.

## 3 RESULTS

### 3.1 Single‐trophic‐level community stability

The SEMs evaluating the influence of species richness, functional diversity, functional composition, species asynchrony and species stability within individual trophic levels on the stability of each trophic‐level community and overall average stability across trophic levels yielded an adequate data fit (Bollen–Stine *p*‐value >0.10 for all SEMs; Figures 1 and 2; Figure S4; Table S4). Analysis of standardized effects, including significant direct and indirect effects from the SEMs, revealed that key predictors of stability differed by trophic level (Figure 1a–c).

**FIGURE 1:**
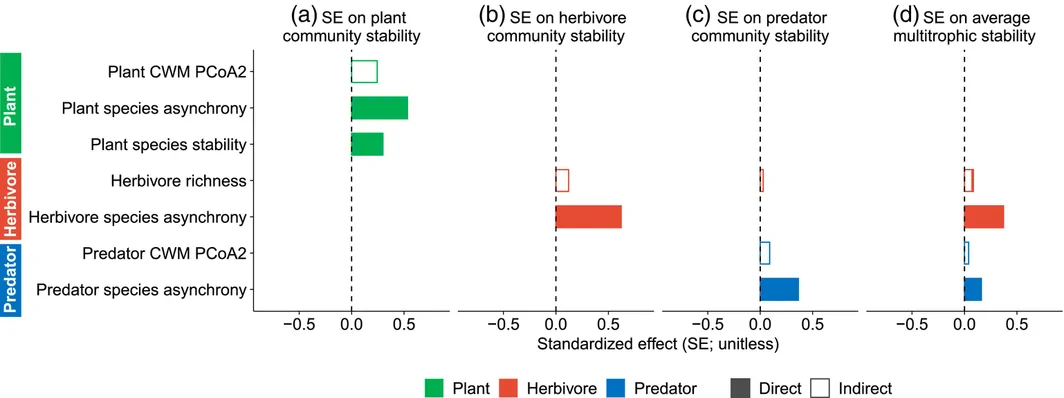
Standardized effects based on structural equation models (SEMs) showing how species richness, functional diversity, functional composition (community‐weighted mean [CWM] trait values), species asynchrony and species stability in plant, herbivore and predator communities are related to (a) plant community stability, (b) herbivore community stability, (c) predator community stability and (d) average multitrophic stability. CWM values for each trophic group were calculated using species coordinates on the first and second axes of the principal coordinate analysis (PCoA) as two synthetic trait axes within the trait space of each group. To avoid multicollinearity, herbivore functional diversity, herbivore CWM PCoA2 and predator CWM PCoA1 were excluded from the analyses. Bars show standardized effects retained in the final SEMs; solid bars indicate significant direct effects; and open bars indicate significant indirect effects. Only significant direct and indirect effects are shown (*p* < 0.05 for all constituent paths). Only variables retained in at least one final SEM are listed on the *y*‐axis; variables without bars in a given panel did not have significant direct or indirect effects in the final SEM for that response variable. See Figure 2, Figure S4 and Table S4 for the corresponding SEM path diagrams and model fit statistics.

**FIGURE 2:**
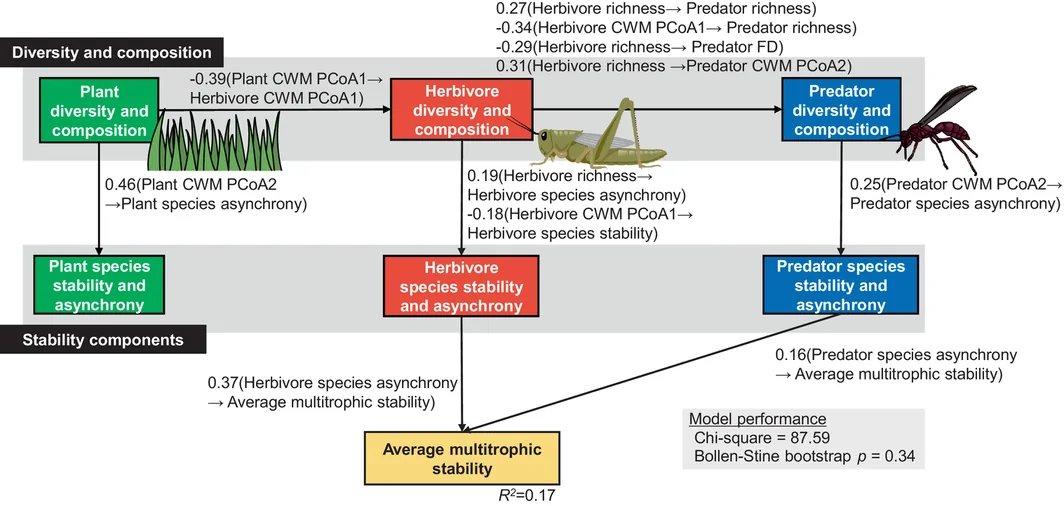
Structural equation model showing how species richness, functional diversity, functional composition (community‐weighted mean trait values; CWMs), species asynchrony and species stability in plant, herbivore and predator communities were related to average multitrophic stability. The CWM values for each trophic group were calculated using species coordinates on the first and second axes of the principal coordinate analysis (PCoA) as two synthetic trait axes within each group's trait space. To avoid multicollinearity, herbivore functional diversity, herbivore CWM PCoA2 and predator CWM PCoA1 were excluded from the analyses. As each box in the figure represents a grouped component rather than a single observed variable, significant fitted relationships are shown as text labels rather than as individual arrows to maintain figure readability. For visual clarity, only significant direct and indirect effects on community stability (*p* < 0.05 for all constituent paths) are present, while correlated errors are omitted. Numbers associated with the text labels indicate standardized regression weights. Model fit metrics (chi‐square and Bollen–Stine bootstrap *p* value) are shown, and *R* ^2^ values denote the proportion of variance explained.

Plant community stability (Figure 1a) was positively associated with plant species asynchrony and species stability. Plant CWM PCoA2 was indirectly and positively associated with plant community stability via plant species asynchrony (Figure 1a; Figure S4a). Higher plant CWM PCoA2 values were associated with lower root nitrogen and phosphorus concentrations, smaller root diameter, smaller leaf area and shorter plant height (Figure S3a), indicating a composite strategy axis combining below‐ground resource‐use traits with plant size‐related variation. Herbivore community stability exhibited a direct positive association with herbivore species asynchrony, while herbivore species richness was indirectly linked to stability via its positive impact on herbivore species asynchrony (Figure 1b; Figure S4b). Predator community stability (Figure 1c) was positively associated directly with predator species asynchrony and indirectly with herbivore species richness and predator CWM PCoA2—defined by decreasing hairiness (Figure S3c). Herbivore species richness was indirectly related to predator community stability via predator CWM PCoA2 and predator species asynchrony, whereas predator CWM PCoA2 was indirectly associated with predator community stability through predator species asynchrony (Figure 1c; Figure S4c).

### 3.2 Average multitrophic stability

Average multitrophic stability demonstrated a positive direct association with herbivore and predator species asynchrony and an indirect association with herbivore species richness and predator CWM PCoA2 (Figures 1d and 2). Herbivore species richness was indirectly related to average multitrophic stability through both herbivore species asynchrony and predator CWM PCoA2, whereas predator CWM PCoA2 exhibited an indirect relationship via predator species asynchrony (Figure 2). Thus, average multitrophic stability shared a subset of the predictors associated with community stability within individual trophic levels, with herbivore species asynchrony exerting the strongest standardized effect, supplemented by contributions from herbivore species richness, predator functional composition and predator species asynchrony.

### 3.3 Threshold‐based multitrophic stability

To complement the averaging approach, we assessed multitrophic stability by counting the trophic‐level stability metrics that exceeded specific thresholds simultaneously (Figure 3; Table S5). Stability among individual trophic levels was not consistently aligned; plant and herbivore community stability showed a negative correlation, whereas other pairwise correlations were non‐significant (Table S6). Hence, relying solely on average multitrophic stability could obscure the distribution of stability across different trophic groups. The threshold‐based analysis facilitated the identification of predictors associated with the simultaneous maintenance of high stability across trophic levels.

**FIGURE 3:**
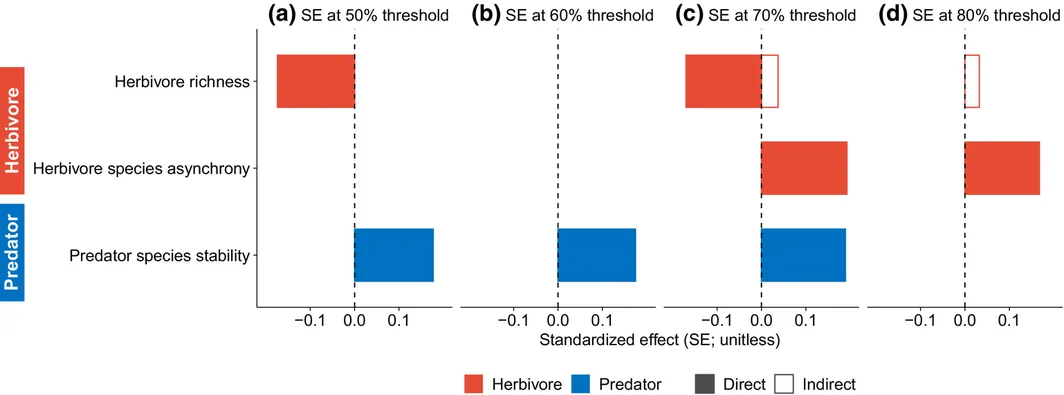
Standardized effects based on structural equation models (SEMs) showing how species richness, functional diversity, functional composition (community‐weighted mean trait values), species asynchrony and species stability in plant, herbivore and predator communities were related to threshold‐based multitrophic stability at the (a) 50%, (b) 60%, (c) 70% and (d) 80% threshold levels. Threshold‐based multitrophic stability was defined as the number of trophic‐level community stability metrics exceeding each threshold. Thresholds are expressed as a percentage of the maximum observed stability at each trophic level, with the maximum calculated as the mean stability value above the 95th percentile. To avoid multicollinearity, herbivore functional diversity, herbivore CWM PCoA2 and predator CWM PCoA1 were excluded from analyses. Bars show standardized effects retained in the final SEMs; solid bars indicate significant direct effects; and open bars indicate significant indirect effects. Only significant direct and indirect effects are shown (*p* < 0.05 for all constituent paths). See Table S5 for model fit statistics.

Herbivore species asynchrony directly increased the number of trophic‐level stability metrics exceeding the threshold at the 70% and 80% levels, suggesting that its positive relationship with multitrophic stability persisted even when high stability was required across all trophic levels (Figure 3c–d). Predator species stability also exhibited positive direct effects at the 50%, 60% and 70% thresholds (Figure 3a–c). Herbivore species richness showed negative direct effects at the 50% and 70% thresholds, but positive indirect effects at the 70% and 80% thresholds (Figure 3a,c,d).

## 4 DISCUSSION

Our study shows that multitrophic stability cannot be comprehensively assessed through a single summary metric. Rather, the two analytical approaches revealed complementary aspects of stability: while the averaging approach provided an aggregate mean measure of multitrophic stability, the multiple threshold approach identified the predictors associated with maintaining consistently high stability across plants, herbivores and predators. Across individual trophic levels, species asynchrony generally emerged as a key determinant of community stability; however, the relative importance of other factors varied among plants, herbivores and predators.

At the multitrophic scale, average stability was associated with herbivore species asynchrony and richness, predator functional composition and predator species asynchrony. Additionally, herbivore species asynchrony was positively associated with sustained high stability across trophic levels according to the multiple threshold approach. Collectively, these results highlight herbivore temporal dynamics—particularly species asynchrony—as the most robust predictor of multitrophic stability, thereby offering empirical evidence for the role of herbivores as key stabilizing components within food webs.

### 4.1 Within‐trophic species asynchrony and community stability

This study revealed that species asynchrony within each trophic group played a central role in stabilizing communities (Figure 1). These findings support our predictions and align with theoretical predictions and empirical evidence from studies focusing on a single trophic level (Caruso et al., 2020; Sasaki et al., 2019; Tilman et al., 2006; Valencia et al., 2020; Wilcox et al., 2017; Yachi & Loreau, 1999). Moreover, they suggest that both increased differentiation in species' responses to environmental changes and compensatory dynamics, whereby declines in certain species are offset by increases in others, enhance the temporal stability of aggregate community properties, such as total biomass and overall abundance.

In our system, plant functional composition was indirectly related to plant community stability via plant species asynchrony, and predator functional composition was indirectly related to predator community stability via predator species asynchrony (Figure S4a,c), whereas herbivore species richness was indirectly associated with herbivore community stability via herbivore species asynchrony (Figure S4b). This suggests that the trait syndromes of dominant species shaped how the most abundant species fluctuated relative to the rest of the community. Because species asynchrony depends on covariance among constituent species, shifts in dominant trait composition can increase asynchrony when dominant species track temporal variation differently from co‐occurring species (de Bello et al., 2021; Loreau & de Mazancourt, 2008). In plants, higher CWM PCoA2 values reflected dominance by species with smaller leaves and thinner, nutrient poorer roots, suggesting that dominant species with this syndrome may respond differently to temporal variation in soil resource conditions than co‐occurring species (Freschet et al., 2021; Lavorel et al., 2011). In predators, CWM PCoA2 primarily reflected lower hairiness, an interaction‐related trait, suggesting that communities dominated by less hairy predators differed in how dominant species tracked prey fluctuations through time (Moretti et al., 2017). Among herbivores, the positive association between species richness and species asynchrony suggests that increased diversity itself may encourage temporal differentiation. Taken together, these insights suggest that species asynchrony is trophic‐level specific, with distinct patterns and drivers across different trophic groups (Firkowski et al., 2022; Liang et al., 2025).

### 4.2 Herbivore species asynchrony and multitrophic stability

The averaging and multiple threshold approaches revealed a consistent finding: herbivore species asynchrony was positively associated with higher average multitrophic stability and the simultaneous maintenance of stability across trophic levels (Figures 1d, 2 and 3). This provides clear empirical evidence that herbivore species asynchrony serves as a robust predictor of multitrophic stability across plants, herbivores and predators, expanding on recent research by Srednick and Swearer (2024) and Liang et al. (2025), by showing in a three‐trophic‐level system how factors associated with community stability within individual trophic levels scale up to shape multitrophic stability.

Our findings highlight the central yet often overlooked influence of herbivores as dynamic intermediaries within trophic networks (Liang et al., 2025; Mortensen et al., 2018; Van Veen & Sanders, 2013). Positioned at an intermediate trophic level, herbivores respond to changes in plant availability and regulate how these fluctuations affect higher trophic levels (Schmitz, 2008; van Veen et al., 2006). The asynchrony among herbivore species may help to buffer fluctuations in plant exploitation and predator provisioning (Van Veen & Sanders, 2013), thus stabilizing energy and biomass flow throughout the food web. This interpretation is consistent with the positive association of herbivore species asynchrony with threshold‐based multitrophic stability at the 70% and 80% levels. At moderate thresholds, the criterion may be met under a broader range of community states, making the number of trophic levels exceeding the threshold less sensitive to the specific buffering role of herbivore species asynchrony. By contrast, at higher thresholds, plots in which asynchronous herbivore dynamics more effectively buffer temporal variation in plant exploitation and predator resource supply are more likely to keep multiple trophic levels above the threshold simultaneously. Previous studies have likewise shown that threshold‐dependent effects often become most apparent under stringent thresholds that capture the concurrent maintenance of high levels of ecosystem functioning, rather than moderate levels (Byrnes et al., 2014; Lefcheck et al., 2015).

Conversely, while predator species asynchrony was also positively correlated with average multitrophic stability, it did not predict the concurrent maintenance of high stability across trophic levels (Figures 1d and 3). This pattern may result from predator communities closely tracking changes in herbivore diversity and abundance (Figure S5), leading to synchronized rather than buffered trophic dynamics (Schmitz, 2008). This difference underscores the importance of temporal buffering at the intermediate‐consumer level to ensure stable multitrophic dynamics.

### 4.3 Herbivore species richness across complementary multitrophic stability metrics

Compared with the consistently positive influence of herbivore species asynchrony, the impact of herbivore species richness on multitrophic stability varies with the stability metric used. Under the averaging approach, herbivore richness showed a positive association with average multitrophic stability, attributable in part to indirect pathways involving predator functional composition and species asynchrony. Conversely, under the multiple threshold approach, herbivore richness exerted negative direct effects at the 50% and 70% thresholds, whereas it exhibited positive indirect effects at the 70% and 80% thresholds. This disparity is plausible, as the two metrics reflect distinct aspects of multitrophic stability (Byrnes et al., 2014). Average multitrophic stability may remain elevated when positive effects on certain trophic levels offset weaker or adverse effects on others, whereas threshold‐based stability assesses whether multiple trophic levels simultaneously achieve high stability.

A plausible explanation for these threshold‐dependent richness effects is that herbivore richness influenced multitrophic stability through two opposing pathways. At moderate to relatively high thresholds, richer herbivore assemblages may amplify temporal fluctuations in plant consumption, thereby reducing plant community stability and generating the negative direct effects observed at the 50% and 70% thresholds (Eschenbrenner & Thébault, 2023; Thébault & Loreau, 2005). At the same time, herbivore richness was positively associated with herbivore species asynchrony, and herbivore species asynchrony was positively associated with threshold‐based multitrophic stability at the 70% and 80% thresholds, indicating a positive indirect pathway under more stringent stability requirements. The 70% threshold appears to represent an intermediate case in which both pathways were detectable simultaneously. At the 80% threshold, however, only the positive indirect pathway remained, suggesting that, under the most stringent criterion considered here, the effect of herbivore richness may be expressed more strongly through herbivore species asynchrony than through the direct destabilizing pathway. Therefore, this pattern does not necessarily contradict the insurance hypothesis (Yachi & Loreau, 1999); rather, it suggests that the net effect of herbivore species richness depends on whether its destabilizing influence on plant community stability or its stabilizing influence through herbivore species asynchrony is more strongly expressed under a given stability criterion. As our SEM identified statistical pathways rather than directly testing consumer–resource interactions, this interpretation remains inferential.

### 4.4 Limitations

While this study provides insights into the drivers of multitrophic stability, some limitations remain, warranting cautious interpretation. First, the analysis is based on only 3 years of data (2017–2019), introducing uncertainty into estimates of stability and species asynchrony and potentially limiting the detection of long‐term compensatory dynamics, delayed responses or rare climatic events. Recent theoretical and empirical work indicates that the strength and direction of species synchrony can shift markedly across different time‐series lengths. For instance, Luo et al. (2025) suggest that competition tends to enhance synchrony over short periods but reduce it over longer durations, resulting in divergent patterns of synchrony and its association with species richness, depending on observation length. This emphasizes the risk of drawing general conclusions about temporal dynamics from short‐term datasets. Hence, our findings should be considered representative of short‐term dynamics rather than general patterns of multitrophic stability across extended temporal scales or broader ecosystem contexts. Longer term observations are necessary to assess the persistence of these patterns over time and under a wider range of environmental conditions.

Second, after approximately 10 years of natural reassembly following the final plant removal manipulations, the plots no longer showed strong, consistent differences in plant diversity or functional composition among treatments (Figure S2). This recovery reduced the strength of the original treatment contrasts, so the present communities are best interpreted as reassembled communities with different treatment histories rather than as communities maintaining strong persistent treatment effects. Accordingly, some observed cross‐trophic associations may reflect variation that emerged during long‐term reassembly or parallel ecological responses to unmeasured environmental fluctuations rather than direct interactions across trophic levels.

Third, while our principal conclusion was supported by two complementary stability metrics, the threshold‐based multitrophic stability approach in this study was based on only three trophic levels, each represented by a single community stability metric. As a result, the response variable could take only four discrete values (0, 1, 2 or 3), which limits the resolution of the threshold‐based metric and may constrain the statistical power and interpretability of the corresponding SEM results. This limitation should be considered when interpreting threshold‐dependent patterns in our analyses. The broader applicability of our results across ecosystem types, functional groups and alternative formulations of multitrophic stability warrants further investigation. It will be crucial for future experiments to use factorial manipulations of biodiversity across several trophic levels and, where possible, incorporate a broader set of community functions or stability dimensions to help clarify causal relationships from co‐varying ecological responses and further elucidate the mechanisms governing multitrophic stability (Liang et al., 2025).

## 5 CONCLUSION

Herbivore species asynchrony is a key predictor of stability when community dynamics are viewed across plants, herbivores and predators. This highlights the intermediate consumer trophic level as a key contributor to temporal buffering within food webs. By systematically incorporating multitrophic time‐series data and diverse stability metrics, this study offers valuable insights into how temporal dynamics within a single trophic group can impact overall multitrophic community stability.

## AUTHOR CONTRIBUTIONS

TS, XL, KU and YB conceived of the study. TS, TT and XL performed data curation. TS, TT, XL, XZ, IN, DM, KT, XS, YI and KU conducted the field survey. TT and YI analysed the data. TS and YB wrote the first draft of the manuscript. All authors contributed substantially to revisions.

## CONFLICT OF INTEREST STATEMENT

The authors declare no competing interests.

## STATEMENT ON INCLUSION

Our study involved collaboration among authors from multiple countries, including researchers based in the country where the study was conducted. All authors were actively involved from the early stages of study design to ensure that the diverse perspectives they represent were integrated from the outset. Where relevant, we cited literature published by scientists from the study region to acknowledge and incorporate local scientific contributions.

## Supporting information


**Figure S1.** A priori structural equation models used to evaluate hypothesized relationships among trophic‐level diversity and composition, stability components and community stability.
**Figure S2.** Variation in plant community properties among plots with different treatment histories during the sampling period (2017–2019).
**Figure S3.** Principal coordinate analysis (PCoA) biplots for the trait spaces of (a) plants, (b) herbivores and (c) predators.
**Figure S4.** Structural equation models showing how species richness, functional diversity, functional composition (community‐weighted mean trait values [CWMs]), species asynchrony and species stability in plant, herbivore and predator communities were related to (a) plant community stability, (b) herbivore community stability and (c) predator community stability.
**Figure S5.** Temporal relationships in species richness and biomass/abundance among plants, herbivores and predators.
**Table S1.** Plant functional group removal treatments under complete and partial removal protocols.
**Table S2.** Plant, herbivore and predator traits used in this study.
**Table S3.** Correlation matrix (Pearson's correlation coefficients) of community stability at individual trophic levels and multitrophic stability among predictors.
**Table S4.** Summary of the structural equation modelling (SEM) used to evaluate how species richness, functional diversity, functional composition (community‐weighted mean trait values), species asynchrony and species stability in plant, herbivore and predator communities are related to plant community stability (Figure S4a), herbivore community stability (Figure S4b), predator community stability (Figure S4c) and average multitrophic stability (Figure 2).
**Table S5.** Summary of the structural equation modelling (SEM) used to evaluate how species richness, functional diversity, functional composition (community‐weighted mean trait values), species asynchrony and species stability in plant, herbivore and predator communities were related to threshold‐based multitrophic stability at the 50% (Figure 3a), 60% (Figure 3b), 70% (Figure 3c) and 80% (Figure 3d) threshold levels.
**Table S6.** Correlation matrix (Pearson's correlation coefficients) of community stability at individual trophic levels.

## Data Availability

Data and code supporting this manuscript are available on figshare (https://doi.org/10.6084/m9.figshare.29203865; Tachibana & Sasaki, 2026).
