# Supplementary material for: Herbivore species asynchrony underpins multitrophic stability across plants, herbivores and predators
Source: J Anim Ecol. 2026 Jul 18;95(10):1970–83. doi: 10.1111/1365-2656.70318 (PMC13643179; doi:10.1111/1365-2656.70318)
Supplement: Supplementary file 1 [file JANE-95-1970-s001.docx]

**Supporting Information**

**Herbivore species asynchrony underpins multitrophic stability across plants, herbivores and predators**

**Takehiro Sasaki*, Taiki Tachibana, Xiaoming Lu, Kei Uchida, Xuezhen Zhao, Issei Nishimura, Daichi Makishima, Kousuke Tachibana, Xi Sun, Yuki Iwachido, Wenhuai Li, and Yongfei Bai***

*** Corresponding authors:** Takehiro Sasaki and Yongfei Bai

E-mail address: sasaki-takehiro-kw@ynu.ac.jp (T. Sasaki), yfbai@ibcas.ac.cn (Y. Bai)

**The file includes:**

Figs. S1 to S5

Tables S1 to S6

**Fig. S1.** A priori structural equation models used to evaluate hypothesized relationships among trophic-level diversity and composition, stability components, and community stability. Models for (a) plant community stability, (b) herbivore community stability, (c) predator community stability, and (d) multitrophic stability, evaluated using the averaging and multiple-threshold approaches. “Diversity and composition” denotes species richness, functional diversity, and functional composition at each trophic level, with functional composition represented by community-weighted mean trait values. “Stability components” denotes species asynchrony and species stability. Arrows indicate hypothesized directional relationships: diversity and composition were allowed to cascade across adjacent trophic levels; within each trophic level, diversity and composition were linked to species asynchrony and species stability; species asynchrony and species stability were linked to focal community stability within the same and adjacent trophic levels; and both diversity/composition variables and stability components were linked to multitrophic stability. Correlated errors were included among all pairs of diversity/composition variables within each trophic level and among all pairs of stability–component variables within the same and adjacent trophic levels, but are omitted from the figure for visual clarity. Multitrophic stability was evaluated using the averaging approach (Maestre et al. 2012) and the multiple-threshold approach (Byrnes et al. 2014).

**Fig. S2.** Variation in plant community properties among plots with different treatment histories during the sampling period (2017–2019). Panels show plot-level mean ± SD values of plant species richness, plant functional diversity (FD), and plant functional composition (CWM PCoA1 and CWM PCoA2) for the (a) complete removal protocol and (b) partial removal protocol. Treatment codes are provided in Table S1. Dashed lines indicate the mean value of each plant community property in the control plots (treatment code 1). Although variation among treatment histories remained evident during 2017–2019, mean differences from the control plots were not significant in linear mixed-effects models with block as a random effect.

**Fig. S3.** Principal coordinate analysis (PCoA) biplots for the trait spaces of (a) plants, (b) herbivores, and (c) predators. See Table S2 for the traits used in the analysis and their abbreviations. Percentages in parentheses indicate the proportion of total variation explained by each axis. For plants, PCoA1 is associated with variation in specific leaf area, leaf nitrogen content, and root carbon content, whereas PCoA2 captures covariation, in this dataset, among fine-root morphology, root nutrient concentrations, and plant size-related traits. For herbivores and predators, PCoA1 is associated with body-size-related traits, whereas PCoA2 is primarily associated with hairiness. Given that each trophic group is ordinated independently, the sign and orientation of PCoA axes are arbitrary; therefore, size-related trait vectors are not necessarily aligned in the same direction within the herbivore and predator ordinations.

**Fig. S4.** Structural equation models showing how species richness, functional diversity, functional composition (community-weighted mean trait values; CWMs), species asynchrony, and species stability in plant, herbivore, and predator communities were related to (a) plant community stability, (b) herbivore community stability, and (c) predator community stability. The CWM values for each trophic group were calculated using species coordinates on the first and second axes of the principal coordinate analysis (PCoA) as two synthetic trait axes within each group's trait space. To avoid multicollinearity, herbivore functional diversity, herbivore CWM PCoA2, and predator CWM PCoA1 were excluded from the analyses. As each box in the figure represents a grouped component rather than a single observed variable, significant fitted relationships are shown as text labels rather than as individual arrows to maintain figure readability. For visual clarity, only significant direct and indirect effects on community stability (*P* < 0.05 for all constituent paths) are present, while correlated errors are omitted. Numbers associated with the text labels indicate standardized regression weights. Model fit metrics (chi-square and Bollen–Stine bootstrap *P* value) are shown, and *R^2^* values denote the proportion of variance explained.

**Fig. S5.** Temporal relationships in species richness and biomass/abundance among plants, herbivores, and predators. Black solid lines and shaded areas indicate significant fits (*P* < 0.05) and 95% confidence intervals for the linear mixed-effects model with random intercepts for plot identity. Positive correlations were observed between herbivore and predator species richness and between herbivore and predator abundance.

**Table S1.** Plant functional group removal treatments under complete and partial removal protocols

| Treatment code | AB | PF | PR | PB | Number of plots |
| --- | --- | --- | --- | --- | --- |
| 1 (Control) |  |  |  |  | 4 |
| 2 | x |  |  |  | 4 |
| 3 |  | x |  |  | 4 |
| 4 |  |  | x |  | 4 |
| 5 |  |  |  | x | 4 |
| 6 | x | x |  |  | 4 |
| 7 | x |  | x |  | 4 |
| 8 | x |  |  | x | 4 |
| 9 |  | x | x |  | 4 |
| 10 |  | x |  | x | 4 |
| 11 |  |  | x | x | 4 |
| 12 | x | x | x |  | 4 |
| 13 | x | x |  | x | 4 |
| 14 | x |  | x | x | 4 |
| 15 |  | x | x | x | 4 |
| 16 | x | x | x | x | 4 |
| Number of removal protocols: 2 (complete and partial removal) | | | | |  |
| Total number of plots | | | | | 128 |

“x” denotes the removal of the plant functional group

**Table S2.** Plant, herbivore, and predator traits used in this study

| Trait | Abbreviation | Trophic group | Data type | Unit |
| --- | --- | --- | --- | --- |
| Specific leaf area | SLA | Plant | Continuous | cm^2^ g^-1^ |
| Leaf area | LA | Plant | Continuous | cm^2^ |
| Leaf dry matter content | LDMC | Plant | Continuous | g g^-1^ |
| Plant height | PH | Plant | Continuous | cm |
| Leaf nitrogen content | LNC | Plant | Continuous | g g^-1^ |
| Leaf carbon content | LCC | Plant | Continuous | g g^-1^ |
| Leaf C:N ratio | LCN | Plant | Continuous | unitless |
| Leaf phosphorus content | LPC | Plant | Continuous | g g^-1^ |
| Specific fine root length | SRL | Plant | Continuous | cm g^-1^ |
| Fine root diameter | RD | Plant | Continuous | cm |
| Root carbon content | RCC | Plant | Continuous | g g^-1^ |
| Root nitrogen content | RNC | Plant | Continuous | g g^-1^ |
| Root C:N ratio | RCN | Plant | Continuous | unitless |
| Root phosphorus content | RPC | Plant | Continuous | g g^-1^ |
| Seed mass | SM | Plant | Continuous | mg |
| Tensile strength | TS | Plant | Continuous | N cm^-1^ |
| Body size | – | Herbivore/predator | Continuous | cm |
| Wing size | – | Herbivore/predator | Continuous | cm |
| Dry weight | – | Herbivore/predator | Continuous | g |
| Body hairiness | – | Herbivore/predator | Discrete (1 to 5) | unitless |

**Table S3.** Correlation matrix (Pearson’s correlation coefficients) among predictors used in the analyses of single-trophic-level and multitrophic stability.

|  | Plant species richness | Plant functional diversity | Plant CWM PCoA1 | Plant CWM PCoA2 | Plant species asynchrony | Plant species stability | Herbivore species richness | Herbivore functional diversity | Herbivore CWM PCoA1 | Herbivore CWM PCoA2 | Herbivore species asynchrony | Herbivore species stability |
| --- | --- | --- | --- | --- | --- | --- | --- | --- | --- | --- | --- | --- |
| Plant species richness |  |  |  |  |  |  |  |  |  |  |  |  |
| Plant functional diversity | 0.414 |  |  |  |  |  |  |  |  |  |  |  |
| Plant CWM PCoA1 | 0.494 | 0.244 |  |  |  |  |  |  |  |  |  |  |
| Plant CWM PCoA2 | 0.251 | 0.337 | 0.523 |  |  |  |  |  |  |  |  |  |
| Plant species asynchrony | 0.179 | 0.255 | 0.331 | 0.458 |  |  |  |  |  |  |  |  |
| Plant species stability | 0.023 | 0.022 | -0.032 | -0.055 | -0.145 |  |  |  |  |  |  |  |
| Herbivore species richness | -0.051 | -0.169 | -0.058 | -0.112 | -0.228 | 0.008 |  |  |  |  |  |  |
| Herbivore functional diversity | 0.127 | 0.079 | 0.257 | 0.078 | 0.073 | -0.074 | 0.073 |  |  |  |  |  |
| Herbivore CWM PCoA1 | -0.121 | -0.078 | -0.386 | -0.171 | -0.142 | 0.063 | 0.001 | **-0.753**** |  |  |  |  |
| Herbivore CWM PCoA2 | -0.150 | -0.184 | -0.408 | -0.221 | -0.223 | 0.006 | 0.106 | -0.462 | **0.822**** |  |  |  |
| Herbivore species asynchrony | -0.071 | -0.019 | -0.186 | -0.099 | -0.214 | 0.157 | 0.194 | -0.009 | -0.041 | -0.030 |  |  |
| Herbivore species stability | 0.007 | 0.119 | 0.043 | 0.082 | 0.182 | 0.028 | -0.041 | 0.095 | -0.122 | -0.121 | 0.109 |  |
| Predator species richness | 0.204 | 0.134 | 0.276 | 0.190 | 0.135 | -0.029 | 0.271 | 0.252 | -0.343 | -0.285 | -0.019 | 0.077 |
| Predator functional diversity | 0.104 | 0.162 | 0.009 | 0.020 | 0.109 | -0.001 | -0.291 | 0.039 | 0.023 | 0.042 | -0.023 | 0.003 |
| Predator CWM PCoA1 | 0.032 | -0.046 | 0.022 | -0.006 | 0.080 | -0.014 | -0.281 | 0.026 | 0.047 | 0.099 | -0.027 | -0.003 |
| Predator CWM PCoA2 | -0.102 | -0.254 | -0.001 | -0.063 | -0.208 | -0.011 | 0.312 | -0.058 | 0.037 | 0.119 | 0.007 | -0.138 |
| Predator species asynchrony | -0.131 | 0.007 | -0.079 | -0.035 | -0.115 | 0.071 | 0.165 | -0.303 | 0.269 | 0.224 | 0.062 | 0.123 |
| Predator species stability | 0.043 | 0.085 | -0.019 | 0.042 | 0.055 | 0.097 | -0.090 | -0.034 | 0.065 | 0.086 | -0.232 | -0.138 |

*(Continued)*

|  | Predator species richness | Predator functional diversity | Predator CWM PCoA1 | Predator CWM PCoA2 | Predator species asynchrony | Predator species stability |
| --- | --- | --- | --- | --- | --- | --- |
| Plant species richness |  |  |  |  |  |  |
| Plant functional diversity |  |  |  |  |  |  |
| Plant CWM PCoA1 |  |  |  |  |  |  |
| Plant CWM PCoA2 |  |  |  |  |  |  |
| Plant species asynchrony |  |  |  |  |  |  |
| Plant species stability |  |  |  |  |  |  |
| Herbivore species richness |  |  |  |  |  |  |
| Herbivore functional diversity |  |  |  |  |  |  |
| Herbivore CWM PCoA1 |  |  |  |  |  |  |
| Herbivore CWM PCoA2 |  |  |  |  |  |  |
| Herbivore species asynchrony |  |  |  |  |  |  |
| Herbivore species stability |  |  |  |  |  |  |
| Predator species richness |  |  |  |  |  |  |
| Predator functional diversity | -0.214 |  |  |  |  |  |
| Predator CWM PCoA1 | -0.379 | **0.777**** |  |  |  |  |
| Predator CWM PCoA2 | 0.076 | -0.597 | -0.442 |  |  |  |
| Predator species asynchrony | -0.015 | -0.201 | -0.155 | 0.247 |  |  |
| Predator species stability | -0.004 | 0.178 | 0.079 | -0.112 | -0.075 |  |

Predictors with high correlations (Pearson’s |*r*| > 0.70, in bold) are excluded to avoid multicollinearity, resulting in the exclusion of FDis and CWM PCoA2 of herbivores, and CWM PCoA1 of predators. Significance levels: ***P* < 0.01.

**Table S4.** Summary of the structural equation modeling (SEM) used to evaluate how species richness, functional diversity, functional composition (community-weighted mean trait values), species asynchrony, and species stability in plant, herbivore, and predator communities are related to plant community stability (Fig. S4a), herbivore community stability (Fig. S4b), predator community stability (Fig. S4c), and average multitrophic stability (Fig. 2). Model fit statistics and *R²* values for the endogenous variables in Figs. S4a–c and 2 are also shown. Significant path coefficients (*P* < 0.05) are shown in bold.

|  | Pathway | Standardized coefficients | | *P* |
| --- | --- | --- | --- | --- |
|  | **Fig. S4a, Chi-square = 96.50, *P* = 0.49** |  | |  |
|  | Plant community stability ← Plant species asynchrony | **0.55** | | **0.000** |
|  | Plant community stability ← Plant species stability | **0.31** | | **0.000** |
|  | **Fig. S4b, Chi-square = 83.36, P = 0.30** |  | |  |
|  | Herbivore community stability ← Herbivore species asynchrony | **0.62** | | **0.000** |
|  | **Fig. S4c, Chi-square = 83.32, P = 0.40** |  | |  |
|  | Predator community stability ← Predator species asynchrony | **0.36** | | **0.000** |
|  | **Fig. 2, Chi-square = 87.59, P = 0.34** |  | |  |
|  | Average multitrophic stability ← Herbivore species asynchrony | **0.37** | | **0.000** |
|  | Average multitrophic stability ← Predator species asynchrony | **0.16** | | **0.046** |
|  | **Identical pathways for Figs. S4a-c and 2** |  | |  |
|  | Plant species asynchrony ← Plant CWM PCoA2 | **0.46** | | **0.000** |
|  | Herbivore CWM PCoA1 ← Plant CWM PCoA1 | **-0.39** | | **0.000** |
|  | Herbivore species asynchrony ← Herbivore richness | **0.19** | | **0.025** |
|  | Herbivore species stability ← Herbivore CWM PCoA1 | **-0.18** | | **0.040** |
|  | Predator richness ← Herbivore richness | **0.27** | | **0.001** |
|  | Predator richness ← Herbivore CWM PCoA1 | **-0.34** | | **0.000** |
|  | Predator FD ← Herbivore richness | **-0.29** | | **0.001** |
|  | Predator CWM PCoA2 ← Herbivore richness | **0.31** | | **0.000** |
|  | Predator species asynchrony ← Predator CWM PCoA2 | **0.25** | | **0.004** |
|  | Plant richness ↔ Plant FD | **0.41** | | **0.000** |
|  | Plant richness ↔ Plant CWM PCoA1 | **0.49** | | **0.000** |
|  | Plant richness ↔ Plant CWM PCoA2 | **0.25** | | **0.006** |
|  | Plant FD ↔ Plant CWM PCoA1 | **0.24** | | **0.007** |
|  | Plant FD ↔ Plant CWM PCoA2 | **0.34** | | **0.000** |
|  | Plant CWM PCoA1 ↔ Plant CWM PCoA2 | **0.52** | | **0.000** |
|  | Predator FD ↔ Predator CWM PCoA2 | **-0.50** | | **0.000** |
|  | ***R^2^* values for the endogenous variables in Figs. S4a–c and 2** | | ***R^2^*** | |
| Plant community stability (Fig. S4a) | | | 0.37 | |
| Herbivore community stability (Fig. S4b) | | | 0.39 | |
| Predator community stability (Fig. S4c) | | | 0.13 | |
| Average multitrophic stability (Fig. 2) | | | 0.17 | |
| Herbivore CWM PCoA1 (identical in Figs. S4a–c and 2) | | | 0.15 | |
| Predator richness (identical in Figs. S4a–c and 2) | | | 0.19 | |
| Predator FD (identical in Figs. S4a–c and 2) | | | 0.09 | |
| Predator CWM PCoA2 (identical in Figs. S4a–c and 2) | | | 0.10 | |
| Plant species asynchrony (identical in Figs. S4a–c and 2) | | | 0.21 | |
| Herbivore species asynchrony (identical in Figs. S4a–c and 2) | | | 0.04 | |
| Herbivore species stability (identical in Figs. S4a–c and 2) | | | 0.03 | |
| Predator species asynchrony (identical in Figs. S4a–c and 2) | | | 0.06 | |

**Table S5.** Summary of the structural equation modeling (SEM) used to evaluate how species richness, functional diversity, functional composition (community-weighted mean trait values), species asynchrony, and species stability in plant, herbivore, and predator communities were related to threshold-based multitrophic stability at the 50% (Fig. 3a), 60% (Fig. 3b), 70% (Fig. 3c), and 80% (Fig. 3d) threshold levels. Threshold-based multitrophic stability was defined as the number of trophic-level community stability metrics exceeding each threshold. Thresholds were expressed as a percentage of the maximum observed stability at each trophic level, where the maximum was calculated as the mean stability value above the 95th percentile. To avoid multicollinearity, herbivore functional diversity, herbivore CWM PCoA2, and predator CWM PCoA1 were excluded from the analyses. Model fit statistics and *R²* values for the endogenous variables in the SEMs at each threshold level are also shown. Significant path coefficients (*P* < 0.05) are shown in bold.

| Pathway | Standardized coefficients | | *P* |
| --- | --- | --- | --- |
| **Fig. 3a, Chi-square = 83.20, *P* = 0.15** |  | |  |
| Plant species asynchrony ← Plant CWM PCoA2 | **0.43** | | **0.000** |
| Herbivore CWM PCoA1 ← Plant CWM PCoA1 | **-0.39** | | **0.000** |
| Herbivore species stability ← Herbivore CWM PCoA1 | **-0.18** | | **0.031** |
| Predator richness ← Herbivore richness | **0.28** | | **0.000** |
| Predator richness ← Herbivore CWM PCoA1 | **-0.33** | | **0.000** |
| Predator FD ← Herbivore richness | **-0.29** | | **0.001** |
| Predator CWM PCoA2 ← Herbivore richness | **0.31** | | **0.000** |
| Predator species asynchrony ← Predator CWM PCoA2 | **0.24** | | **0.004** |
| Multitrophic stability count ← Herbivore richness | **-0.17** | | **0.041** |
| Multitrophic stability count ← Predator species stability | **0.18** | | **0.034** |
| **Fig. 3b, Chi-square = 77.17, P = 0.16** |  | |  |
| Plant species asynchrony ← Plant CWM PCoA2 | **0.43** | | **0.000** |
| Herbivore CWM PCoA1 ← Plant CWM PCoA1 | **-0.39** | | **0.000** |
| Herbivore species asynchrony ← Herbivore richness | **0.18** | | **0.034** |
| Herbivore species stability ← Herbivore CWM PCoA1 | **-0.19** | | **0.027** |
| Predator richness ← Herbivore richness | **0.28** | | **0.000** |
| Predator richness ← Herbivore CWM PCoA1 | **-0.34** | | **0.000** |
| Predator FD ← Herbivore richness | **-0.30** | | **0.000** |
| Predator CWM PCoA2 ← Herbivore richness | **0.31** | | **0.000** |
| Predator species asynchrony ← Predator CWM PCoA2 | **0.28** | | **0.000** |
| Multitrophic stability count ← Predator species stability | **0.17** | | **0.038** |
| **Fig. 3c, Chi-square = 81.58, P = 0.16** |  | |  |
| Plant species asynchrony ← Plant CWM PCoA2 | **0.44** | | **0.000** |
| Herbivore CWM PCoA1 ← Plant CWM PCoA1 | **-0.39** | | **0.000** |
| Herbivore species asynchrony ← Herbivore richness | **0.19** | | **0.025** |
| Herbivore species stability ← Herbivore CWM PCoA1 | **-0.19** | | **0.023** |
| Predator richness ← Herbivore richness | **0.29** | | **0.000** |
| Predator richness ← Herbivore CWM PCoA1 | **-0.34** | | **0.000** |
| Predator FD ← Herbivore richness | **-0.29** | | **0.001** |
| Predator CWM PCoA2 ← Herbivore richness | **0.31** | | **0.000** |
| Predator species asynchrony ← Predator CWM PCoA2 | **0.25** | | **0.003** |
| Multitrophic stability count ← Herbivore richness | **-0.17** | | **0.046** |
| Multitrophic stability count ← Herbivore species asynchrony | **0.19** | | **0.023** |
| Multitrophic stability count ← Predator species stability | **0.19** | | **0.023** |
| **Fig. 3d, Chi-square = 76.15, P = 0.18** |  | |  |
| Plant species asynchrony ← Plant CWM PCoA2 | **0.44** | | **0.000** |
| Plant species stability ← Plant CWM PCoA2 | **-0.18** | | **0.039** |
| Herbivore CWM PCoA1 ← Plant CWM PCoA1 | **-0.39** | | **0.000** |
| Herbivore species asynchrony ← Herbivore richness | **0.19** | | **0.025** |
| Herbivore species stability ← Herbivore CWM PCoA1 | **-0.20** | | **0.021** |
| Predator richness ← Herbivore richness | **0.29** | | **0.000** |
| Predator richness ← Herbivore CWM PCoA1 | **-0.35** | | **0.000** |
| Predator FD ← Herbivore richness | **-0.28** | | **0.001** |
| Predator CWM PCoA2 ← Herbivore richness | **0.31** | | **0.000** |
| Predator species asynchrony ← Predator CWM PCoA2 | **0.25** | | **0.003** |
| Multitrophic stability count ← Herbivore species asynchrony | **0.17** | | **0.048** |
| *R^2^* values for the endogenous variables in Fig. 3a–d | | *R^2^* | |
| Plant species asynchrony (Fig. 3a) | | 0.183 | |
| Herbivore CWM PCoA1 (Fig. 3a) | | 0.149 | |
| Herbivore species stability (Fig. 3a) | | 0.034 | |
| Predator richness (Fig. 3a) | | 0.187 | |
| Predator FD (Fig. 3a) | | 0.081 | |
| Predator CWM PCoA2 (Fig. 3a) | | 0.097 | |
| Predator species asynchrony (Fig. 3a) | | 0.059 | |
| Multitrophic stability count (Fig. 3a) | | 0.063 | |
| Plant species asynchrony (Fig. 3b) | | 0.187 | |
| Herbivore CWM PCoA1 (Fig. 3b) | | 0.149 | |
| Herbivore species asynchrony (Fig. 3b) | | 0.032 | |
| Herbivore species stability (Fig. 3b) | | 0.036 | |
| Predator richness (Fig. 3b) | | 0.193 | |
| Predator FD (Fig. 3b) | | 0.089 | |
| Predator CWM PCoA2 (Fig. 3b) | | 0.097 | |
| Predator species asynchrony (Fig. 3b) | | 0.061 | |
| Multitrophic stability count (Fig. 3b) | | 0.030 | |
| Plant species asynchrony (Fig. 3c) | | 0.194 | |
| Herbivore CWM PCoA1 (Fig. 3c) | | 0.149 | |
| Herbivore species asynchrony (Fig. 3c) | | 0.038 | |
| Herbivore species stability (Fig. 3c) | | 0.037 | |
| Predator richness (Fig. 3c) | | 0.194 | |
| Predator FD (Fig. 3c) | | 0.082 | |
| Predator CWM PCoA2 (Fig. 3c) | | 0.097 | |
| Predator species asynchrony (Fig. 3c) | | 0.062 | |
| Multitrophic stability count (Fig. 3c) | | 0.090 | |
| Plant species asynchrony (Fig. 3d) | | 0.192 | |
| Plant species stability (Fig. 3d) | | 0.031 | |
| Herbivore CWM PCoA1 (Fig. 3d) | | 0.149 | |
| Herbivore species asynchrony (Fig. 3d) | | 0.038 | |
| Herbivore species stability (Fig. 3d) | | 0.039 | |
| Predator richness (Fig. 3d) | | 0.206 | |
| Predator FD (Fig. 3d) | | 0.081 | |
| Predator CWM PCoA2 (Fig. 3d) | | 0.097 | |
| Predator species asynchrony (Fig. 3d) | | 0.063 | |
| Multitrophic stability count (Fig. 3d) | | 0.028 | |

**Table S6.** Correlation matrix (Pearson’s correlation coefficients) of community stability at individual trophic levels

|  | Plant community stability | Herbivore community stability | Predator community stability |
| --- | --- | --- | --- |
| Plant community stability |  |  |  |
| Herbivore community stability | **-0.173*** |  |  |
| Predator community stability | -0.020 | 0.004 |  |

Significance level: **P* < 0.05.
